# In-line high resolution PET and 3T MRI hybrid device for preclinical multimodal imaging

**DOI:** 10.1186/2197-7364-1-S1-A7

**Published:** 2014-07-29

**Authors:** Juan J Vaquero, Paula Montesinos, Lorena Cussó, Veronica García-Vazquez, Manuel Desco

**Affiliations:** Departamento de Bioingeniería e Ingeniería Aeroespacial, Universidad Carlos III de Madrid, Kragujevac, Spain; Instituto de Investigación Sanitaria Gregorio Marañón, Madrid, Spain

In this work we evaluate a combination of a high-performance PET with a superconductive 3T MRI system. This PET/MRI system is implemented in a tandem configuration similar to some scanners used in clinical imaging but with both subsystems placed in proximity, what allows sharing a common animal holder that travels between both FOVs.

The PET detector consists in two rings, each one having 18 DOI detectors, which provide a high sensitivity with a 1.0mm^3^ resolution at the centre and minimal degradation toward the edges of the 68mm diameter FOV.

The MRI system is a compact, cryogen-free, auto-shielded and with a very tight fringe-field and no special room requirements. These characteristics enable the close location of the PET detector without deteriorating its performance.

We have evaluated the system obtaining images of three mice in cardiology, oncology and neurology applications. All PET scans were performed 30m after intraperitoneal FDG injection of 25.2±5.8MBq and were reconstructed using a 3D-OSEM algorithm. For the cardiac MRI a gated FLASH sequence and a body coil were used. Tumor MRI was using an FSE T1w sequence and a body coil. Finally, brain MRI was done with an FSE T2w sequence.

In all the cases registration of PET and MRI images was obtained following an equivalent protocol to the standard PET/CT preclinical system, and since the animals didn’t move from the bed the results were satisfactory. Attenuation correction was not applied to any image. The preliminary results from this prototype suggest that the tandem solution for preclinical PET/MRI is feasible and has the advantage of preserving the best image quality in both modalities without requiring any trade-off of specifications. The limitation of not being able to perform simultaneous acquisitions is compensated by the high quality results of studies that do not actually require such simultaneous data collection.

